# Movement induced tremor in musicians and non-musicians reflects adaptive brain plasticity

**DOI:** 10.3389/fpsyg.2014.00824

**Published:** 2014-07-29

**Authors:** André Lee, Erwin Schoonderwaldt, Mareike Chadde, Eckart Altenmüller

**Affiliations:** ^1^Institute for Music Physiology and Musicians’ Medicine, University of Music, Drama and Media HannoverHannover, Germany; ^2^Hannover Medical UniversityHannover, Germany

**Keywords:** physiological tremor, inhibition, EMD, Hilbert spectrum, musician, dystonic tremor, dystonia

## Abstract

Evidence exists that motor dexterity is associated with a higher tremor amplitude of physiological tremor. Likewise, lower frequencies are associated with motor control. So far only case reports of a higher amplitude of physiological tremor in musicians exist. Moreover, no study has investigated lower frequencies during a finger movement task in musicians who can be regarded as a model of motor expertise. We developed a model and derived three hypotheses which we investigated in this study: (1) Tremor amplitude is higher in the range of physiological tremor and (2) higher for frequency ranges of dystonic tremor in musicians compared to non-musicians; (3) there is no difference in tremor amplitude at frequencies below 4 Hz. We measured tremor during a finger flexion-extension movement in 19 musicians (age 26.5 ± 8.2 years) and 24 age matched non-musicians (age 26.5 ± 8.7). By using empirical mode decomposition in combination with a Hilbert transform we obtained the instantaneous frequency and amplitude, allowing to compare tremor amplitudes throughout the movement at various frequency ranges. We found a significantly higher tremor amplitude in musicians for physiological tremor and a tendency toward a higher amplitude during most of the movement in the frequency range of 4–8 Hz, which, however, was not significant. No difference was found in the frequency range below 4 Hz for the flexion and for almost the entire extension movement. Our results corroborate findings that the 8–12 Hz oscillatory activity plays a role in motor dexterity. However, our results do not allow for the conclusion that tremor at the frequency range of 4–8 Hz is related to either plasticity induced changes that are beneficial for motor skill development nor to maladaptive changes as, e.g., focal dystonia.

## INTRODUCTION

Tremor is one of the most common movement disorders, characterized by an involuntary, oscillatory and rhythmic movement with a heterogeneous etiology. Physiological tremor is a normally occurring limb oscillation at a low amplitude (Deuschl et al., 1998) usually within a frequency range of 8–12 Hz (Elble, 1986; Hallett, 1998; Deuschl et al., 2001). Its origin is multifactorial and includes mechanical properties of the limb (Deuschl et al., 1998; Hallett, 1998; McAuley and Marsden, 2000), stretch reflex components (Hagbarth and Young, 1979; Deuschl et al., 1998; McAuley and Marsden, 2000), cardioballistic properties (Elble and Randall, 1978) as well as central oscillators (McAuley et al., 1997; Deuschl et al., 1998; McAuley and Marsden, 2000; Raethjen et al., 2000; Williams et al., 2010). Rather than being merely biological noise, it is thought to play a role in motor unit synchronization (McAuley and Marsden, 2000). Furthermore a recent study by Deutsch et al. (2011) could show a correlation between motor dexterity and an increase in amplitude in the 6–12 Hz frequency band that was more pronounced for movement-related tremor than for postural tremor. It has been shown that finger movements are not smooth but characterized by discontinuities within the frequency range of physiological tremor of 8–10 Hz that occur at different finger velocities (Vallbo and Wessberg, 1993). These discontinuities were discussed to be due to an oscillatory central motor command for finger movements, rather than due to either short or long–latency reflex mechanisms. Supporting evidence for this notion was found by Williams et al. (2009) who could show a significant interaction between the primary motor cortex and the peripheral oscillations. The description of a higher power of the 8–10 Hz discontinuities during finger flexion-extension movements of a cellist with a high level of hand dexterity as compared to a non-musician who self-reported poor manual skills (Vallbo and Wessberg, 1993), as well as the report of a postgraduate piano student with enhanced physiological tremor (Walsh, 1995) are noteworthy, since they are indicative that a correlation may exist between motor skills and physiological tremor. To our knowledge, however, differences in tremor amplitude of physiological tremor between musicians and non-musicians have not been systematically investigated so far in a task that involves a flexion-extension movement of single fingers, which is one of the most essential movements for the performance of most instruments.

**FIGURE 1:**
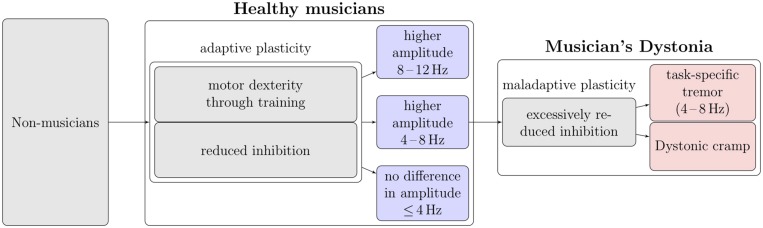
**Model of training induced changes in musicians.** The three predictions tested in this study are shown as arrows to the blue boxes. It is known that intense practice leads to enhanced motor dexterity, which itself is associated with an increase in amplitude at the 8–12 Hz frequency range (Deutsch et al., 2011). Thus a higher amplitude at this frequency range should be expected (hypothesis 1). Furthermore it is known that in musicians inhibition is reduced (Rosenkranz et al., 2005) which may cause dystonia if a further reduction occurs (Chen et al., 1997; Rosenkranz et al., 2005). One manifestation of dystonia are task-specific tremors (Deuschl, 2003; Gironell and Kulisevsky, 2009; Elble and Deuschl, 2011) for which a reduced inhibition has been shown (Byrnes et al., 2005). The frequency range in task-specific tremor in musicians was found to be in the 3–8 Hz frequency range (Lee et al., 2013b). Thus plasticity induced “dystonogenic” changes may possibly be detectable at the peripheral level as well as a subclinical increase in amplitude at the frequency range of 4–8 Hz. Subclinical means that even though tremor amplitude is higher, it does not (yet) interfere with playing ability (hypothesis 2). If the reasoning for hypothesis 2 were true, no change in tremor amplitude should be expected at frequencies not associated with dystonic tremor (<4 Hz; hypothesis 3).

A reduced intracortical inhibition was shown in healthy musicians (Nordstrom and Butler, 2002; Rosenkranz et al., 2005), that was discussed as being beneficial for the process of improving fine motor skills while learning an instrument. However, if inhibition is further reduced it may lead to musicians (Rosenkranz et al., 2005). In the context of dystonia, task-specific tremors have been discussed as dystonic tremors (Deuschl, 2003; Gironell and Kulisevsky, 2009; Elble and Deuschl, 2011) and described in musicians at a frequency range of 3–8 Hz (Lee et al., 2013a,b). The pathophysiology of dystonic tremor DT is thought to underlie similar mechanisms as those leading to dystonia (Deuschl and Bergman, 2002; McAuley and Rothwell, 2004) like a reduced inhibition at different levels of the nervous system (Nakashima et al., 1989; Chen et al., 1997; Berardelli et al., 1998; Lin and Hallett, 2009). One study could show a reduced inhibition in primary writing tremor (Byrnes et al., 2005). We therefore hypothesized that brain alterations due to extensive practice of fine motor skills may manifest themselves at a peripheral level as a subclinical increase in tremor amplitude at the frequency range of 4–8 Hz where subclinical means that tremor is not (yet) impairing playing ability of the musician. Rather it has been suggested that it may be related to motor control, as well (Gross et al., 2002). However, it is possible that it becomes a clinically relevant task-specific tremor or task-specific dystonia (Rosenkranz et al., 2005) if reduced inhibition on a central level is pathologically reduced. Interestingly, no study assessed tremor amplitude at lower frequencies in musicians so far. We therefore developed a model that takes into consideration these observations (**Figure 1**).

The aim of the study was to investigate three hypotheses or predictions derived from this model: (1) based on the findings by (Deutsch et al., 2011) we expected a significantly higher tremor amplitude at a frequency range of 8–12 Hz in healthy musicians compared with non-musicians during slow finger movements. (2) If reduced inhibition in healthy musicians may be regarded as a precursor of musician’s dystonia that may progress to overt dystonia (Rosenkranz et al., 2005) or task-specific tremor as a form of dystonic tremor (Deuschl, 2003; Gironell and Kulisevsky, 2009; Elble and Deuschl, 2011), a subclinically higher tremor amplitude in the frequency range of 4–8 Hz in musicians is detectable. Subclinically means that tremor does not interfere with instrument playing. (3) Following hypothesis (2), there is no difference in tremor amplitude at frequencies not associated with dystonia, i.e., <4 Hz.

## MATERIALS AND METHODS

### PARTICIPANTS

We measured fingers II–V of both hands in 19 healthy professional musicians as the experimental group (mean age 26.5 ± 8.2 years). Musicians had started playing their instrument at an age of 7.2 ± 2.9 years and had 19.3 ± 7.1 years of training. The time practiced per day was 3.5 ± 2.0 h. Twelve musicians (63.2%) played the piano, three (15.8%) played the guitar and a string instrument, respectively, and one (5.3%) played the flute (**Table 1**). Two musicians were left-handed. We included 24 healthy non-musicians matched for age and handedness as a control group (age 26.5 ± 8.7). Age difference was not statistically significant (Wilcoxon signed rank test: *W* = 255.5, *p* = 0.5).

**Table 1:** Musicians’ characteristics.

	Mean	SD
Age (yrs)	26.5	8.2
Age when starting the instrument (yrs)	7.2	2.9
Average practice time per day (hours)	3.5	2.0
Years of training	19.3	7.1

	***n***	**%**

**Gender**		
Female	7.0	36.8
Male	12.0	63.2
**Instrumental group**		
Keyboard	12	63.2
Guitar	3	15.8
String	3	15.8
Woodwind	1	5.3

*Abbreviations: yrs, years; SD, standard deviation.*

### MEASUREMENT

Tremor was measured with a 3D accelerometer (biovision, Wehrheim, Germany, 8 × 8 × 11 mm; 4 g; DC – 500 Hz; max 50 g) and Ag-AgCl-surface-EMG (biovision, Wehrheim, Germany). The accelerometer was placed on the fingernail of the finger to be measured. For examination patients were seated in a comfortable chair with the hand palm facing upward and the forearm placed on a comfortable armrest. Participants were instructed not to hold the arm against gravity, since it is known that this may significantly increase tremor at the frequency range of 8–12 Hz (Morrison and Sosnoff, 2009). Furthermore, we instructed the participants to relax the contralateral arm to avoid cross modulation of tremor (Chen et al., 2011). Participants were asked to exert a flexion-extension movement of digits II–V of both hands with each finger being measured separately and starting from an extended position of the respective finger. The flexion movement was such that the distal phalanx described an angle of 180°. Each direction had a duration of 4 s and was paced by a metronome set to 60 bpm. Thus, one cycle of flexion-extension lasted for 8 s. Fingers II–V were measured separately for 3.5 m each in a randomized order. Data of digits II–V of one hand had to be removed for one musician and one non-musician due to technical problems. We chose to assess a movement-induced tremor rather than postural tremor for the following reasons: firstly, a higher postural tremor may not be expected from someone who is skilled in a fine motor task (Chen et al., 2011); secondly because it has been shown that training-induced increase of physiological tremor is more prominent during movement than during a static condition (Deutsch et al., 2011); and finally because it has been suggested that postural tasks are related to a 20 Hz oscillatory activity (McAuley et al., 1997), which might have an influence on physiological tremor.

## DATA PROCESSING

**FIGURE 2:**
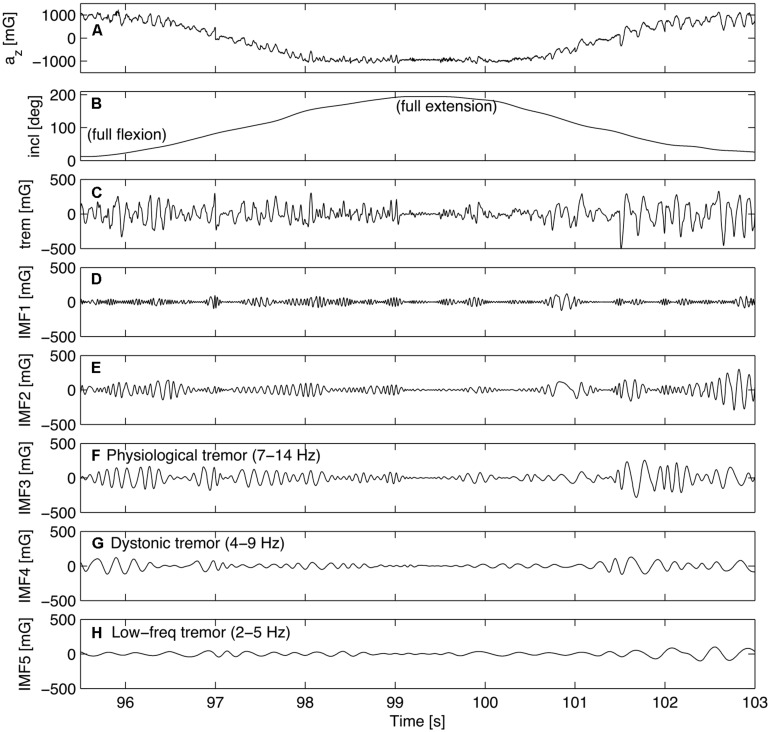
**Example of the decomposition of the tremor signal of a single complete movement cycle (flexion-extension) of the left ring finger of one musician.** The panels give an overview of the different stages of signal processing: **(A)** the z-component of the calibrated accelerometer signal (raw data), **(B)** finger inclination angle (voluntary flexion-extension movement), **(C)** band-pass filtered accelerometer signal (tremor), **(D–H)** intrinsic mode functions (IMFs) 1–5, representing decreasing frequency ranges. The association with specific types of tremor is indicated in the respective panels. Acceleration and amplitude values are expressed in units of milligravity (mG).

It is known that tremor is a non-linear, non-stationary process (Gantert et al., 1992; Elble, 1996; Deuschl et al., 1998). We therefore applied empirical mode decomposition (EMD; Huang et al., 1998), a data-driven digital signal processing technique that is suited for non-linear and non-stationary signals and may distinguish voluntary movement from tremor (De Lima et al., 2006; Gallego et al., 2011). In a sifting process, signals are decomposed into basic components, called intrinsic mode functions (IMF; **Figure 2**) in order to identify frequency bands (De Lima et al., 2006; Silchenko et al., 2010; Li et al., 2012; Lee et al., 2013a) that may be related to biological phenomena like tremor. This property was of special interest, since we were interested in three particular frequency bands (see introduction).

With regard to the accelerometer signal, the inclination angle of the finger was calculated using a four-quadrant inverse tangent of the z- and the *y*-axis components. A low-pass filter (2 Hz) was applied to filter the voluntary part of the finger movement. From the *z*-axis the tremor signal was obtained by applying a 4th order butterworth band-pass filter (1–50 Hz) back and forth to obtain zero phase shift. Next, EMD was performed in Matlab using the EMD package by Rilling, (2007)^1^, applying the default stopping criterion (Hogan, 1984). Finally, the Hilbert transform was applied to the IMFs to obtain the instantaneous frequency and amplitude (Hilbert-spectrum). For an overview see **Figure 2**.

**FIGURE 3:**
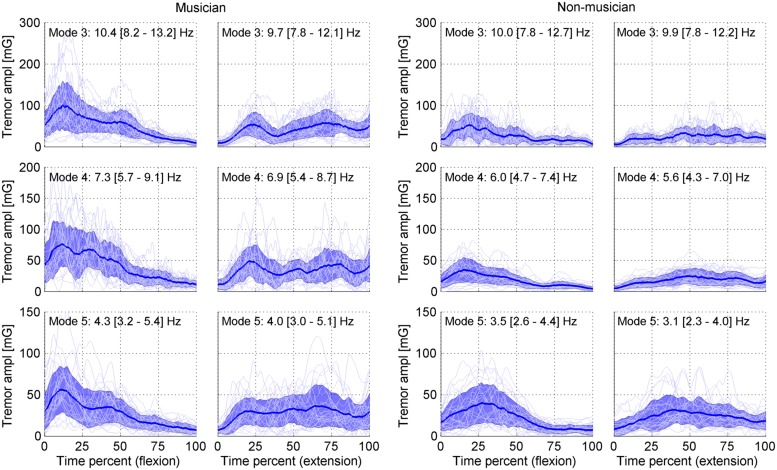
**Tremor-amplitude-curve distribution of IMF 3–5 (modes 3–5) in the left index finger of one musician (left panel) and one non-musician (right panels).** The left graph of each panel shows the flexion movement from maximal extension to maximal flexion. The right graph shows the extension movement from maximal flexion to maximal extension. The *y*-axis shows the tremor amplitude in units of (mG). The *x*-axis shows time as a percentage of total flexion and extension duration, respectively, (about 4 s each). The bold line shows the mean amplitude across cycles within the trial (3.5 min.), and the shaded area indicates the standard deviation. The thin lines show the amplitude curves of the individual cycles. The frequency range of the modes (mean, maximum and minimum frequency) is indicated by the text above the graphs. The irregular tremor distribution over the course of the movement for both directions is visible, which is indicative for the non-stationarity of the tremor signal.

For statistical analysis of the amplitude curves associated with the IMFs each flexion and extension part of the movement (half cycle of about 4 s duration) was resampled to a standard time grid. The sample-rate roughly corresponded to that of the original signal (1840 samples per half cycle). The movement reversals (flexion to extension and *vice versa*) were identified from the inclination signal. Finally, average amplitude curves for each movement direction were calculated for each finger for selected IMFs (**Figure 3**).

For further evaluation IMFs 3–5 were chosen because IMF 3 contained the frequency band of physiological tremor, IMF 4 the frequency band of dystonic or task-specific tremor in musicians and IMF 5 lower frequencies (**Figure 3**). For our comparisons we calculated *differences* between amplitudes curves. Since the true distribution of our difference curves was not known we applied a bootstrapping procedure to calculate the 95% confidence interval (CI). By considering difference curves, significant differences become apparent when zero lies outside the CI.

Difference curves and their respective CI were constructed as follows:

(1) For each musician/non-musician pair an average difference curve was calculated, matched with respect to hand (L/R) and finger (II–V), i.e., across a maximum of eight curves per pair, since eight fingers were investigated. By matching with respect to hand and finger, possible differences of tremor amplitude between different fingers were controlled for. This yielded a total of 456 difference curves (19 musicians × 24 non-musicians).
(2) The overall difference curve and CI were estimated by means of an iterative bootstrapping procedure. Within each iteration, a bootstrap sample of observations (i.e., musicians) was constructed by sampling with replacement. The bootstrap sample size was kept equal to the original number of observations (*N* = 19). To each observation within the bootstrap sample one of the 24 control participants was randomly assigned, and the corresponding difference curves were selected. Then the sample’s average difference curve was calculated and stored. After 10,000 iterations the overall difference curve and its one-sided 95% CI was calculated by taking the average and the fifth percentile across iterations.

## RESULTS

**FIGURE 4:**
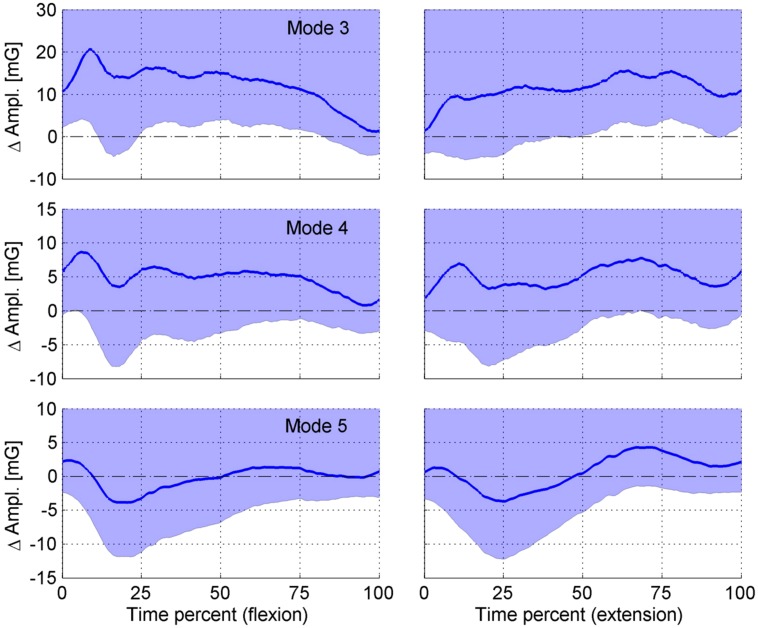
**Difference in tremor-amplitude between the fingers of musicians (M) and the non-musicians (NM) for IMFs (modes) 3–5.** The left graphs show the flexion movement (from an extended finger to a flexed finger) and the right graphs the extension movement (from the flexed finger to an extended finger). The *x*-axis shows time as a percentage of total flexion and extension duration, respectively (about 4 s each). The *y*-axis shows the tremor amplitude difference between musicians and non-musicians in units of (mG). The thin dashed line at ΔAmpl = 0 is the zero reference line for the comparisons. A positive value of ΔAmpl indicates a higher tremor amplitude in the musician group. The shaded areas show the bootstrapped one-sided 95% confidence intervals (CI) for the comparisons.

For our hypotheses we compared the difference in tremor amplitude between musicians and non-musicians of IMF 3–5 (**Figure 4**).

Hypothesis 1: The comparison at the frequency range of 8–12 Hz revealed a significantly higher amplitude throughout almost the entire flexion except at the beginning and the end of the movement and the second half of the extension movement in the frequency range of 8–12 Hz.
Hypothesis 2: Mean amplitude was higher throughout both movement directions in the 4–8 Hz frequency range, however, statistical significance was not reached.
Hypothesis 3: In the frequency range <4 Hz there was no difference in the mean amplitude for the flexion movement and lower at the beginning and higher at the end of the extension movement. However, the differences were not statistically significant.

## DISCUSSION

The aim of the study was to test three hypotheses or predictions derived from a model (**Figure 1**) in which we integrated findings of studies on tremor and its relation to motor skill acquisition as well as to malplasticity caused by excessive training-leading to dystonia or task-specific tremor. We are aware that general conclusions must be taken cautiously and remain speculative. Our hypotheses did not include the question, whether in musicians a difference exists between hands that are more involved in motor control (e.g., left hand in string players, right hand in piano players) and the contralateral hand. Neither did we investigate, whether the handedness plays a role. However, future studies should investigate hypotheses derived from these questions.

### HYPOTHESIS 1

A recent study investigated the progression of the 6–12 Hz oscillatory drive during childhood development under a static condition (isometric wrist extension against gravity) and a dynamic condition (wrist flexion-extension) by assessing the coherence between EMG-signals and the accelerometer signal. They found an increasing coherence in the 6–12 Hz frequency with increasing age under both, the static and the dynamic condition, with a significantly higher increase of coherence for the dynamic condition and a positive correlation between the increase in coherence and motor dexterity (Deutsch et al., 2011). However, in that study motor dexterity was measured with a sequential finger to thumb opposition task, which did not involve the wrist movement subsequently measured during the experiment. In the present study we therefore measured tremor in a finger movement task, since this is an extensively trained movement for most musicians. There is evidence that independence of movements across finger is important for precisely timed movement sequences (Fuglevand, 2011) as making music (Furuya et al., 2011) and it has been shown that musicians have a higher independence of movements across fingers as compared to non-musicians (Slobounov et al., 2002; Aoki et al., 2005). To achieve the high level of expertise necessitates more than 10,000 h of intense practice during childhood and adolescence (Ericsson et al., 1993). The first hypothesis was thus that musicians exhibit a higher tremor amplitude in the 8–12 Hz frequency range when performing a finger flexion-extension movement. Tremor amplitude was higher throughout the entire movement and reached statistical significance for a great part of the flexion and the second half of the extension movement. This finding corroborates the suggestion that 8–12 Hz oscillations “may contribute to improvements in movement efficiency, execution timing and speed” (Deutsch et al., 2011). The fact that statistical significance was not reached throughout the entire movement to our point of view reflects the fact that the effect was too small for the sample size measured. In a review by McAuley and Marsden (2000) it was suggested that at the peripheral level an oscillatory output enhances a more linear output thereby helping to “overcome inertial resistances at movement onset” (Greene, 1972). Furthermore, the role of tremor in timing was stressed and the advantages of a pulsatile motor output over a continuous output discussed (Welsh and Llinás, 1997). Interestingly, a 80–100 ms (i.e., 10–12.5 Hz) interval is the time needed for a feedback-guided reaction to motor actions (Falkenstein et al., 1990; Johansson, 1998). Thus a discontinuous impulse may not only decrease computational demands as opposed to a continuous command (McAuley and Marsden, 2000) but may increase efficiency in feedback-driven motor commands necessary for error correction. This notion is supported by functional neuroimaging studies that could show that musicians use motor networks including areas for motor control more efficiently than non-musicians (Haslinger et al., 2004). It is known that functional changes in the brain are associated with musical training. Rosenkranz et al. (2005) could show a reduced intracortical inhibition in healthy musicians (Nordstrom and Butler, 2002), that was discussed as being beneficial for the process of learning an instrument. Indeed, reduced inhibition may lead to a facilitation of the 10 Hz central oscillations, leading to an improvement in temporospatial precision.

### HYPOTHESIS 2

Mean tremor amplitude was higher throughout the entire movement at the frequency range of 4–8 Hz where pathological task-specific tremor in musicians (Lee et al., 2013b) can be found. However, this difference reflected a trend and did not reach statistical significance. The rationale behind this study were findings that lower frequencies may play a role in movement control, as suggested in a study by Gross et al. (2002). We therefore hypothesized that a reduction of intracortical inhibition beneficial for skill acquirement in healthy musicians that may lead to musicians dystonia if it progresses (Rosenkranz et al., 2005) may be detectable as an increased tremor amplitude in tremulous activity in frequency ranges associated with dystonic or task-specific tremor (i.e., 4–8 Hz; Deuschl, 2003; Gironell and Kulisevsky, 2009; Elble and Deuschl, 2011; Lee et al., 2013a,b). This increase was to be subclinical, not interfering with music making but deteriorating, if a further maladaptive reduction of inhibition occurs. Although a trend toward a higher amplitude was visible, the small effect makes a relationship to task-specific tremor unlikely. Still, the question remains, why task-specific tremors manifest themselves in lower frequency ranges and not in a frequency range of 8–12 Hz, where reduced inhibition leads to an increase in amplitude. A possible, albeit speculative, explanation may be the finding of an antiphase oscillation of 10 Hz at the spinal level which dampens tremor amplitude in healthy persons at this frequency range (Williams et al., 2010). Thus an increase in central motor output at this frequency range may be compensated for, whereas no such mechanism seems to exist at lower frequency ranges. Thus, an increase in oscillatory movement at the affected limbs at lower frequency ranges cannot be compensated for.

### HYPOTHESIS 3

In frequency ranges below 4 Hz almost no difference was seen during the flexion movement and a lower amplitude for the first half of the extension movement in the extension movement. This corroborates the notion that oscillations at frequencies below 4 Hz do not seem to play a role in motor dexterity.

## CONCLUSION

We proposed a model that integrated recent findings of tremor and its role in motor dexterity as well as its relation to maladaptive plasticity, leading to dystonia or task-specific tremor. However, the second hypothesis derived from the model could not be statistically confirmed. Our findings therefore corroborate the notion that physiological tremor is related to motor dexterity through intense training (Deutsch et al., 2011) and manifests itself at the peripheral level as an increased tremor amplitude in musicians as compared to non-musicians. However, from our findings we cannot conclude that the increase in tremor amplitudes in the frequency range of 4–8 Hz are related to motor skill development or to maladaptive plasticity.

## AUTHOR CONTRIBUTIONS

Eckart Altenmüller and André Lee were substantial contributors to the conception and design of the work. André Lee and Mareike Chadde were substantial contributors to the acquisition and Erwin Schoonderwaldt, André Lee and Mareike Chadde to the analysis and interpretation of the data. André Lee was drafting the work and Erwin Schoonderwaldt, Eckart Altenmüller and Mareike Chadde were revising it critically for important intellectual content. All authors gave their final approval of the version to be published and agreed to be accountable for all aspects of the work in ensuring that questions related to the accuracy or integrity of any part of the work are appropriately investigated and resolved.

## Conflict of Interest Statement

The authors declare that the research was conducted in the absence of any commercial or financial relationships that could be construed as a potential conflict of interest.

## Footnotes

1 http://perso.ens-lyon.fr/patrick.flandrin/emd.html
